# Is preterm birth a human-specific syndrome?

**DOI:** 10.1093/emph/eov010

**Published:** 2015-06-14

**Authors:** Julie Baker Phillips, Patrick Abbot, Antonis Rokas

**Affiliations:** Department of Biological Sciences, Vanderbilt University, VU Station B 35-1364, Nashville, TN 37235, USA

**Keywords:** prematurity, fitness, gestation length, allometry

## Abstract

Human preterm birth (PTB), a multifactorial syndrome affecting offspring born before 37 completed weeks of gestation, is the leading cause of newborn death worldwide. Remarkably, the degree to which early parturition contributes to mortality in other placental mammals remains unclear. To gain insights on whether PTB is a human-specific syndrome, we examined within- and between-species variation in gestation length across placental mammals and the impact of early parturition on offspring fitness. Within species, gestation length is normally distributed, and all species appear to occasionally give birth before the ‘optimal’ time. Furthermore, human gestation length, like that of many mammalian species, scales proportionally to body mass, suggesting that this trait, like many others, is constrained by body size. Premature humans suffer from numerous cognitive impairments, but little is known of cognitive impairments in other placental mammals. Human gestation differs in the timing of the ‘brain growth spurt’, where unlike many mammals, including closely related primates, the trajectory of human brain growth directly overlaps with the parturition time window. Thus, although all mammals experience early parturition, the fitness costs imposed by the cognitive impairments may be unique to our species. Describing PTB broadly in mammals opens avenues for comparative studies on the physiological and genetic regulators of birth timing as well as the development of new mammalian models of the disease.

## I. INTRODUCTION

Preterm birth (PTB), defined in humans as birth before 37 completed weeks of gestation, is a complex multifactorial syndrome that originates when the complex interplay of ‘anatomical, physiological, biochemical, endocrinological and immunological events’ necessary for parturition is disrupted [1]. Complications of PTB are the leading cause of death in newborns and in children under the age of 5 [2]. Globally more than 1 in 10 babies is born before 37 weeks of gestation, and PTB rates appear to be increasing in almost all countries [3]. PTB can stem from environmental factors such as infection, inflammation and stress [1], as well as genetic ones; for example, familial studies have demonstrated an increased risk for PTB in women with sisters who have given birth prematurely [4] and in women whose grandparents were born preterm [5]. More recently, genome-wide association studies have begun to identify candidate genes associated with increased risk of PTB in various human populations [1, 6].

A complete understanding of PTB will require identifying the molecular and genetic mechanisms that control gestation length, pregnancy maintenance and initiation of parturition. Although the study of animal models has yielded many insights into the physiology of pregnancy, the functional mechanisms that result in early parturition remain poorly understood [7–9]. This is so in part to our lack of understanding of whether the syndrome of PTB is human-specific, or whether other organisms experience PTB as well. Although to our knowledge this question has not been heretofore explicitly addressed, experts have so far argued for the uniqueness of human parturition [10, 11]. If, as Smith has stated, ‘human parturition is a distinctly human event’ [10] then it follows that pathologies of parturition, such as PTB, might also be confined to our species. Several statements in the published literature seem to support this notion with respect to PTB [12–15]. For example, the 2007 National Academies report on PTB stated that ‘most animal species do not have significant rates of spontaneous preterm birth’ [15], Rubens *et al.* recently opined that ‘spontaneous idiopathic PTB is very uncommon in species other than humans’ [12], and Bryant-Greenwood *et al.* stated that ‘the serious clinical problem of spontaneous PTB appears to be almost unknown in species other than humans’ [14].

If indeed PTB is unique to humans, then considerable nuance is required in translating studies of animal models to an understanding of the etiology of PTB, as presumably most of the genetic contributors to the disease evolved after the divergence of humans from the chimps, e.g. [6, 16, 17]. However, if PTB is not restricted to humans, knowledge on the prevalence and symptoms of PTB in placental mammals has the potential to invigorate research strategies through comparative studies on the physiological and genetic regulators of birth timing as well as the development of new mammalian models of the disease.

Addressing the degree to which PTB is a human-specific syndrome requires carefully examining three questions. First, is the amount of variation in gestation length of humans distinct from that of other mammals? Additionally, is there large enough variation in birth timing, as there is in humans, such that some births normally occur before full-term gestation in other mammals? Second, is the average gestation length of human pregnancy in any way unusual compared to other mammals? Answering this question is directly relevant to addressing whether human gestation length has been uniquely influenced by selection. For example, it has been recently argued that PTB is more common in humans due to selection for shortened gestation length driven by fitness costs associated with cephalopelvic disproportion [6]. Finally, if mammals broadly experience PTB, what is the impact of variation in gestation length on offspring fitness across the species that exhibit it? In this critical review, we address the first question by surveying the distributions of gestational lengths and the second question through examining the evolutionary constraints on gestation lengths relative to body size and brain size across a wide range of placental mammals. To address the third question, we examine the fitness costs associated with early parturition in humans and other mammals. Finally, we discuss how the answers to these questions provide a novel evolutionary perspective to studying the molecular basis of PTB broadly in mammals.

## II. GESTATION LENGTH SHOWS SIMILAR INTRA-SPECIES VARIATION ACROSS MAMMALS

**Figure 1.**
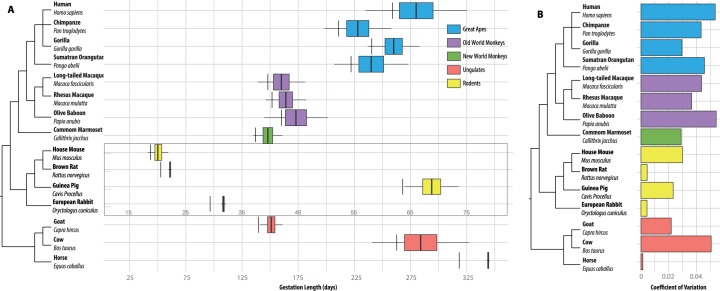
Intra-species variation in gestation length is similar among many mammals. (A) We collected the arithmetic means and standard deviations, when available, in days for all placental mammals with complete genomes. Sample sizes ranged from 2 to 17 000, with a median of 104. In all cases, only live births were considered. Examination of the potential for skew in model choice (normal vs log-normal) showed that the mean squared error between the two distributions was likely well below the error in measurement of gestation lengths reported in the original research. Boxes contain the mean plus/minus 1 SD; whiskers extend to plus/minus 3 SD. Vertical lines indicate 92.5% completed gestation time suggesting each species experiences ‘preterm’ birth according to the human definition with the exclusion of horses, goats and rodents. (B) Comparison of the coefficient of variation across species. Plots and analysis were performed using the ggplot2 package in R 3.1.2 [122, 123]. References for each species can be found as follows: human [25], chimpanzee [29], gorilla [124], orangutan [125], long-tailed macaque and rhesus macaque [126], baboon [127], marmoset [128], rat and rabbit [129], guinea pig [24], goat and mouse [130], cow [131] and horse [132]

All quantitative traits, such as human height [18–20], have a continuous range of variation. In this context, we expect the presence of measurable variance in reproductive traits. For example, long-term population studies in British islands, such as with Soay sheep in St Kilda island and red-tailed deer on the Isle of Rum, have uncovered abundant variation in longevity, age at primiparity, lifetime fecundity and lifetime reproductive success [21–23], and have provided strong evidence for the heritability of such variation [21, 22]. For example, the average lifetime fecundity in the Ram Mountain population of Soay sheep is 5.3 lambs, ranging from 0 to 15, and its estimated heritability is significantly larger than zero, suggesting that this reproductive trait is not only variable, but that its variance has a heritable component [23]. The insights we have gained from these long-term studies on variation in reproductive traits suggest that heritable variation in gestation length, like other reproductive traits, should also be a general feature of mammalian life history. Indeed, gestation length data from diverse placental mammals show that all experience variation in birth timing (Fig. 1). For example, guinea pigs, *Cavia porcellus*, have gestation lengths ranging from 8.5 to 10 weeks [24], whereas humans have gestation lengths ranging from 28 to 50 weeks [25]. From Fig. 1, we see there is no expectation from life-history theory that gestation length variation is absent in mammals generally, nor is there evidence that variation in human gestation length is remarkable or unusual compared to other mammal species, particularly other primates.

## III. PTB IS OBSERVED IN MANY MAMMALS

Although all mammals experience variation in gestation length, is the syndrome of PTB itself unique to humans? The World Health Organization defines human PTB as ‘babies born alive before 37 weeks of pregnancy are completed’ [26]. Contributed by the World Health Organization and supported by the International Federation of Gynecology and Obstetrics, the basis of this definition stems from a statistical analysis of the distribution of gestational age at birth, based on the first day of the last menstrual period [27]. The purpose of such a definition was to provide a standardized language for PTB, but the definition lacks medical or biological meaning; as a result ‘preterm’ should be distinguished from ‘premature’, which describes a lack of completed fetal development [28].

Although no definition of PTB exists for species other than our own, it is interesting to contemplate how the gestational length-based definition of human PTB applies to other species. For example, generalizing the human-based definition of PTB as ‘parturition prior to 92.5% (259 days or 37 weeks/280 days or 40 weeks) completed gestation’ and assuming that gestational length is a normally distributed variable [25] allows us to examine the occurrence of PTB in any placental mammal species for which population gestational length data are available.

Applying this generalized, percentage-based cut-off definition of PTB to gestation length data from diverse species shows that parturition before 92.5% completed gestation occurs in many organisms, including in all examined primates, such as chimpanzees and gorillas (Fig. 1). Interestingly, one definition of PTB in chimpanzees, defined as 2 SD below the mean [29], results in an estimated 16% of chimpanzees born preterm, suggesting that the prevalence of ‘PTB’ in chimpanzees, our closest relatives, appears to be similar to the prevalence of PTB in humans [2]. Although it is generally unclear whether PTB is spontaneous or induced in these animals, evidence from horses suggests that PTB in non-human mammals can result from placental infections [30], a well-documented cause of PTB in humans [31].

Interestingly, ‘PTB’ appears to be absent in most of the animal models of the human syndrome. The gestation lengths of newborns in mice, rats, and guinea pigs do not appear to cross below a threshold of 92.5% of completed gestation (Fig. 1). The apparent absence of PTB in Rodentia is particularly interesting as this order shows remarkable diversity in both gestation length, which can range from 20 days (e.g. *Mus musculus*) to 150 days (e.g. *Hydrochoerus hydrochaeris*), and developmental strategy; young may be precocial (e.g. *Mus musculus*) or altricial (e.g. *Cavia porcellus*). This diversity suggests that the mechanisms underlying the apparent absence of PTB in this lineage may not be influenced by gestation length or by developmental strategy.

## IV. OPTIMAL GESTATIONAL LENGTH SCALES WITH BODY SIZE

Allometry, namely how traits scale with one another [32], has long provided a framework for not only understanding how traits function and vary across ecological and evolutionary time, but also for identifying outliers and the underlying ecological or evolutionary reasons that gave rise to them [33]. Allometric studies have been used extensively to predict the values of morphological, ecological, and physiological traits as a function of an organism's body size, as measured by body mass [34–41]. Well-known examples of allometric traits that scale with body mass include vertebrate brain size [35, 39], longevity [38, 40] and basal metabolic rate [34, 36, 37, 41]. For example, both the basal metabolic rate and brain size scale with mammalian body mass to the three-fourth of the power [37, 42].

**Figure 2.**
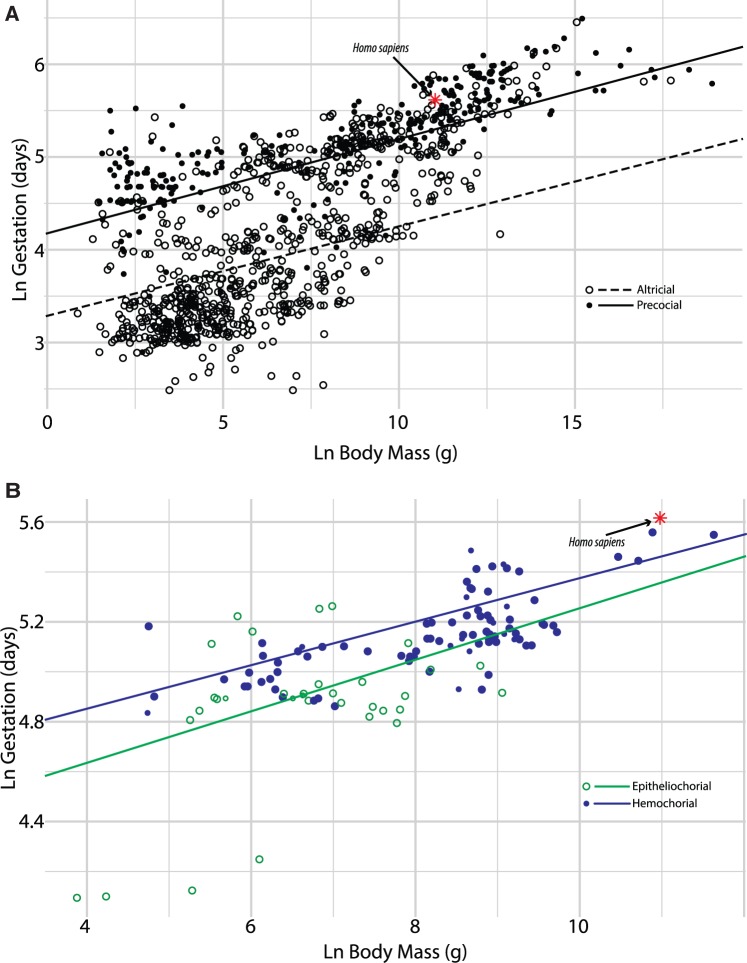
Gestation length is constrained by maternal body mass in placental mammals. Logarithmic plot of gestation length (days) against maternal body mass (grams) for 1100 placental mammals (A) and 120 primates (B). The scaling coefficient for all mammals is 0.09 (SE = 0.007). Altricial and precocial mammals have similar slopes, 0.10 (SE = 0.008) and 0.10 (SE = 0.01), respectively. Within primates, the scaling coefficient is 0.08 (SE = 0.02). Epitheliochorial and hemochorial have similar slopes, 0.10 (SE = 0.04) and 0.09 (SE = 0.02), respectively. Mass and gestation length data taken from the PanTheria database [133]. Offspring number per litter was used as a proxy for neonate development state. Placental structure was as described by Mossman [134]. Data were linked to a supertree of extant mammals [135]. We present the relationship between log-transformed body mass, and log-transformed gestation length using phylogenetic generalized least squares. Statistical tests were performed in R 3.1.2 [123] using the packages ape [136], caper [137] and nlme [138]

Allometric relationships have also been described for many mammalian reproductive traits, such as litter weight [38, 43, 44], neonate weight [38, 43–45], neonate brain weight [42, 43, 46] and the per capita growth rate (Malthusian parameter) [38, 45], providing a window for understanding the evolution of pregnancy-associated traits in mammalian species and the identification of trends and constraints. For example, study of the relationship between neonatal brain mass and body size has identified an evolutionary trend toward larger brain size relative to fetal body mass compared to non-primates [42]. Gestation length has also been found to scale to maternal body mass by 1/4 [38, 43, 47, 48], but subsequent studies utilizing phylogeny-informed statistics support a scaling exponent closer to 0.10 [49, 50] (Fig. 2). The relationship between body mass and gestation length suggests that the timing of gestation in mammals is either constrained by maternal body mass, or that the two traits are under a shared constraint. For example, recent work has suggested that human gestation length may be primarily constrained by metabolism [51], raising the alternative hypothesis that gestation length and maternal body mass, which also allometrically scales with metabolism, may be under a shared metabolic constraint.

The diversity of traits associated with mammalian reproduction and pregnancy may also play an important role in controlling gestation length. For example, mammals employ different precocial and altricial strategies in neonate development state at birth. Precocial species have offspring that are typically well-developed, born with eyes open and are immediately mobile (e.g. most ungulates). In contrast, offspring from altricial species are born while relatively immobile, deaf, blind and unable to obtain food without parental assistance. Precocial mammals typically have longer gestation periods compared to altricial ones. When a distinction is made between altricial and precocial mammals, the scaling relationships of precocial and altricial mammals become distinct, but retain similar scaling exponents [48, 50] (Fig. 2). Additionally, offspring in altricial mammals are typically born in litters; increased litter sizes have been shown to reduce gestation lengths in cats [52] and dogs [53]. Similarly, multiple births in humans show reduced gestation lengths, with 50% of twin and 90% of triplets pregnancies born preterm [54].

Another pregnancy-associated phenotype that varies extensively across mammals is placental morphology. Mammalian placentas can be classified into three principle types of interfaces, namely epitheliochorial, endotheliochorial and hemochorial. Frequently more than one placental interface occurs within an order, e.g. epitheliochorial in strepsirrhine primates and hemochorial in haplorrhine primates. Placental shape and interdigitation also vary frequently within mammalian orders. Interestingly, placental structure correlated with differences in gestation length and fetal growth [55]. Analysis of gestation length against body mass in haplorrhine primates, which all have hemochorial placentas, shows that humans are typical for organisms with hemochorial placentas (Fig. 2).

Human encephalization has resulted in a cephalopelvic disproportion that has been argued to play a role in the complication of labor [56], and that the dramatic cephalopelvic changes have resulted in shortened gestation lengths in humans [6]. Encephalization is not a primate trait but rather a complex combination of changes in brain growth specific to the human lineage [57]. Humans and closely related primates do not share a common brain ontology [57], thus calculating human gestation by comparing brain size is not sufficient evidence to suggest a shortening of gestation length in humans. To make allometric analyses from an obviously enlarged feature would be akin to suggesting that human skeletal mass is decreased relative to brain size, when studies have demonstrated a reliable allometric relationship between body and skeletal mass [58].

Characterization of allometric relationships also provides the opportunity to identify outliers: organisms in which the relationship between two biological variables of interest significantly deviates from the majority of others. Such outliers can be informative about evolution, as they reflect cases in which one of the variables has been sculpted by adaptive evolution. For example, primates tend to have larger brain sizes than predicted by a scaling relationship between brain size and body mass in mammals. Within primates, humans have a brain size 3.1 times larger than predicted for the average primate [59]. For gestation length, organisms with longer gestation lengths than expected given their body mass are primarily from the order Chiroptera, whereas organisms with shorter than expected gestation lengths are largely from Cetacea [47]. Mammals in these orders may be ideal for comparative and functional genomic analyses to better understand regulation of gestation timing.

In summary, optimal gestation length in most mammals appears to be strongly correlated with body size; simply put, for most mammals, including humans, gestation length can be easily be extrapolated from their body mass. This correlation is rather surprising, not only given that many of the reproduction- and pregnancy-associated traits discussed above vary widely across mammals but also given the genetic complexity stemming from the interplay of maternal, paternal and fetal genomes inherent in mammalian pregnancy [60]. Thus, even though control of parturition, whether maternal, fetal or both, has been shown to be an important regulator of gestation length and birth timing [61–64], it appears that such molecular control mechanisms of parturition are evolutionarily coupled with organism body size.

## V. PREMATURITY IMPOSES FITNESS CONSEQUENCES

Fitness is a complex measure that accounts for numerous life-history traits in age-structured populations. Fitness has been equated with reproductive success [65, 66], and for the purpose of evolutionary genetics, fitness measures the rate of increase in individuals possessing specific genotypes or phenotypes [67]. Individuals with increased rates of survival and reproductive success are expected to have increased fitness [66, 68].

Discussing the fitness consequences of PTB requires that we first disentangle the meanings of ‘preterm’, which denotes an earlier than expected timing of parturition and the quantity that most human PTB studies rely on, and ‘premature’, which denotes a lack of completed fetal development and the source of any fitness consequences associated with PTB [28]. Because PTB is defined by gestational age in humans, it is often divided into three subcategories: extremely preterm (birth before 28 weeks completed gestation), very preterm (birth before 32 weeks completed gestation, but after 28) and moderate/late preterm (birth before 37 weeks completed gestation, but after 32). As each sub-category has associated complications and levels of prematurity [69], fitness differentials between preterm and full-term offspring can be interpolated by the differential survival rates of each group.

Neonate mortality is lowest in infants born at full-term, between 38 and 41 weeks of gestation, with mortality rates rising inversely to gestational age in preterm infants [70, 71]. The significant impact of PTB on offspring fitness is indicated by the fact that PTB is the leading direct cause of neonate mortality (defined as deaths within the first 4 weeks of life) worldwide; for example, approximately 27% of the 4 million neonatal deaths in 2000 were attributed to complications from PTB [72]. Preterm infants have higher rates of cerebral palsy [73–75], chronic lung disease [76–78], necrotizing enterocolitis [79–81], retinopathy [82–84], hearing impairments [75] and hospital readmissions [85, 86] compared to full-term infants. Neonate deaths and increased rates of chronic health conditions arise from immature organ systems that are not yet developed to support life outside the intrauterine environment [15].

In the few records of fitness outcomes for PTB in primates, both chimpanzees and pigtail macaques experience decreased survival rates resulting from preterm delivery [29, 87]. In chimpanzees, all but one chimpanzee in 17 recorded preterm deliveries (≤208 days as defined by Wildman *et al.*) were aborted, stillborn or died during the neonatal period [29]. In pigtail macaques, greater than 95% of ‘high risk newborns’, which include premature, low birth weight, and maternally rejected offspring, die if left in maternal care. In contrast, if provided care in a nursery environment, the mortality rate of high risk newborn pigtail macaques is reduced to only 20%. Premature pigtail macaques have not only decreased survival but complex patterns of behavioral traits that differ from full-term offspring [87].

The fitness consequences of PTB continue to be highly noticeable in early childhood [73, 88, 89], with complications from PTB being the largest cause of mortality in children under 5 [2]. For example, mortality rates in early childhood, ages 1–5, in a large Swedish cohort not only showed a strong, significant inverse relationship with gestational age [90], and thus reduced fitness compared to full-term children, but also a significant association between decreasing gestational age and the severity of the fitness cost.

Fewer studies have examined the long-term effects of prematurity in young adults [90–96]. Mortality rates in young adulthood, ages 18–36, also show an inverse relationship with gestational age [90, 96]. The outcomes of extremely low birth weight (ELBW) infants, whose average gestational age was 27.1 weeks, include a substantially larger number of incidents of neurosensory impairments (NSI) including cerebral palsy, mental retardation, blindness and deafness, and were more likely to include multiple impairments [96]. Additionally, male ELBW infants have increased prevalence of physical conditions including seizures, asthma and recurrent bronchitis [92, 95, 96]. Even though the prevalence of NSI was higher in young adults with low birth weights, studies support that young adults born with low birth weights are only slightly disadvantaged in regards to participation in sports and other social activities, as well as romantic and sexual relationships [92, 95, 97, 98]. The argument that preterm born adults have similar quality of life is surrounded by dissention both in the medical community [97, 98] and by parents [99, 100]. The precise degree to which fitness is affected in preterm infants that survive to adulthood, especially those impacted by NSI, remains unclear.

Defining PTB by a percentage based cut-off leads to the inference that many placental mammals experience preterm delivery. However, understanding the impact, if any, of variation in gestation length on offspring fitness in non-human mammals is challenging due to the lack of studies [12, 29]. One area where the relationship between fitness and parameters associated with reproduction has been well studied in both humans and other mammals [101–104] is in the context of changes in the environment. In humans, offspring born during the Finnish famine experienced decreased survival rates, but the increased mortality rates did not persist in later life [104] suggesting the fitness differential due to maternal exposure to environmental hardships lessens once the offspring reaches reproductive age. In wild mammal populations, differences in birth weights of red deer are associated with seasonal temperature fluctuations during the final months of gestation; lower birth weights in cooler temperatures are linked to decreased neonatal survival and increased age at first reproduction [101]. In Soay sheep, increased population density, which probably leads to competition for limited food resources and increased competition for mates, is associated with reduced birth weights and neonatal survival [102].

In summary, the synthesis of the currently available data suggests that although the fitness consequences of PTB in the early years of human life are very large in infancy and early childhood, the consequences may be smaller in adulthood. Furthermore, NSI are the most consistently reported difference between preterm and full term neonates. Cognitive deficits, in the absence of major motor defects, are the dominant neurodevelopmental sequelae in PTB infants [105]. The degree to which human-specific or human-elaborated adaptations contribute to these cognitive deficits is unknown. Although little data exist on either the rate of incidence of prematurity in other mammals or the resulting cognitive impairments of PTB in other mammals, the results to date indicate that neonate fitness is linked to gestational age, environmental fluctuations and reduced birth weights [29, 87, 101, 102].

## VI. TIMING OF BRAIN DEVELOPMENT MAY PLAY AN IMPORTANT ROLE IN HUMAN PREMATURITY

One obvious difference between humans and our closely related primates is our highly increased immaturity at term birth, primarily due to the substantial postnatal brain growth required for normal human development [56, 107]. The ‘brain growth spurt’ defines the window during development when the brain is passing through its most rapid period of growth [108, 109], and can be visualized as a sigmoidal curve when brain growth is plotted against age [110]. Rough categorization of growth spurts suggests three categories: (i) prenatal, (ii) perinatal and (iii) postnatal. Altricial young undergo growth spurts prenatally, precocial young postnatally, whereas organisms that exhibit intermediates between altriciality and precociality undergo growth spurts perinatally. For example, the perinatal growth spurt in humans places the species in the intermediate state of development, previously described as ‘secondarily altricial’ [111], which is evidenced by the typically singleton births of neonates with open eyes and ears at birth (precociality) combined with relative helplessness of human babies compared to other primates (altriciality) [56, 107] and by the reduced neonate brain size relative to the adult size in humans compared to chimps [112].

Importantly, the brain growth spurt time window not only reflects a period where brain size grows dramatically, but also a period of enhanced vulnerability of the growing brain and organism to endogenous and exogenous insults [105, 108, 109, 113]. Humans experience the most rapid brain growth during the perinatal and into the postnatal phase of development [113, 114], but recent comparison to chimpanzees has shown our closest extant relative does not share this pattern of brain growth [114]. Rapid brain growth in chimpanzees continues to approximately 22 weeks of the 34–35 week gestation period, whereas rapid human brain growth continues to at least 32 weeks [114]. Therefore, in a manner distinct from our closest primate relatives, the period of enhanced vulnerability resulting from the brain growth spurt overlaps with parturition in humans.

**Figure 3.**
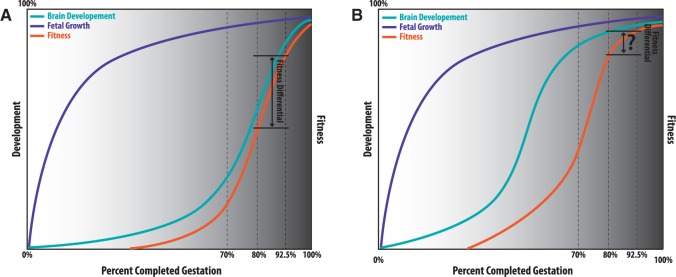
Preterm birth in humans may result in greater fitness differential compared to non-human primates. Normal fetal growth (purple) is similar between humans (A) and non-human primates (B). Unlike fetal growth, humans experience a growth spurt in brain development (teal) later in gestational time compared to non-human primates. As a result, the fitness (orange) differential between a pre-term human born at 80% completed gestation and a human born at 92.5% completed gestation could be substantially larger than that of non-human primates

Brain growth patterns are highly variable in closely related primates [115]; a more complete understanding of the variation in these patterns is necessary to better understand cognitive impairments that result from PTB. The vulnerability of the brain due to rapid growth rates at parturition may play an important role in the cognitive impairments resulting from early parturition in humans, and as such this may explain the lack of cognitive impairments in organisms with ‘growth spurts’ primarily occurring during prenatal or postnatal development (Fig. 3). Recent work in baboons has provided evidence that the sequence of cerebral development and pattern of cerebral injury between the prematurely delivered baboons is remarkably similar to that of prematurely born humans [116], but the long-term behavioral phenotypes have yet to be described in this promising animal model.

## VII. THE SIGNIFICANCE OF AN EVOLUTIONARY PERSPECTIVE FOR UNDERSTANDING HUMAN PTB

Gestation length is normally distributed and scales proportionally to body mass across a wide diversity of placental mammals, suggesting that not only is this trait correlated with body size, but also that many mammals give birth before the ‘optimal’ time. Thus, humans are not unique in the variation or in the length of gestation relative to other mammals. Prematurely born humans suffer numerous NSI, but knowledge of cognitive impairments in other placental mammals is lacking because studies are rare and difficult, given the drastic mortality rate of PTB in mammals outside of humans. The timing of the human ‘brain growth spurt’ has the potential to explain increased cognitive impairments in premature humans as the trajectory of growth is unlike closely related primates and directly overlaps with the parturition time window.

What guidance, if any, does this evolutionary perspective provide for furthering our understanding of the molecular basis of PTB? We believe that this critical review illustrates three ways, which have generally not been considered in the PTB literature, to advance our understanding of this serious syndrome.

First, the widespread occurrence of PTB in wild mammal populations strongly argues for decoupling preterm parturition from premature parturition and suggests that all mammals could in principle be useful, at least through comparative and functional genomics experiments, for understanding gestation length and birth timing, even if they are poor models for understanding the pathogenesis of the syndrome. In fact, it can be argued that a mechanistic understanding of the regulation of mammalian gestation length would in fact contribute to understanding PTB pathogenesis, albeit not by direct inference.

Second, the significant deviations in the allometric relationships between gestation length and body mass of organisms in the orders Chiroptera and Cetacea relative to the relationships observed in most other mammals, raises the hypothesis that the evolution of these two traits (gestation length and body mass) might be less correlated or decoupled in these two orders. Much like species with exaggerated or novel characters, which have been exploited by evolutionary developmental biologists to generate insights into the genetic basis of the underlying characters [117, 118], species in these two orders can be viewed as outliers that harbor great promise for beginning to elucidate the molecular mechanisms that control mammalian gestation length and timing.

Third, a comparative perspective across development at the tissue level provides a way to identify organisms that better model disease aspects of the PTB syndrome. For example, the resemblance between the lung histopathology of premature lambs to that of chronic lung diseases in preterm infants has led to the development of lambs as a model for bronchopulmonary dysplasia [119]. Similarly, the brain growth spurt in pigs, like humans, spans the prenatal, perinatal and postnatal development, leading to suggestions that it has potential as an appropriate model for human infant brain development [120, 121]. Finally, the presence of similar patterns of cerebral injury in premature baboons and humans [116] suggests that non-human primates may be useful models of NSI that result from human PTB.

In summary, we have made clear that placental mammals experience ‘non-optimal’ birth timing and that early parturition results in fitness costs through increased mortality in both human and non-human primates, but the fitness cost of prematurity in survivors remains elusive. The combination of brain growth timing as well as the secondarily altricial nature of human offspring may be features that make human parturition unique to experience PTB as a syndrome of complications, but continued comparative studies in gestation length, birth timing and brain development may reveal additional similarities between humans and other placental mammals.
